# Salidroside promotes peripheral nerve regeneration based on tissue engineering strategy using Schwann cells and PLGA: *in vitro* and *in vivo*

**DOI:** 10.1038/srep39869

**Published:** 2017-01-05

**Authors:** Hui Liu, Peizhen Lv, Yongjia Zhu, Huayu Wu, Kun Zhang, Fuben Xu, Li Zheng, Jinmin Zhao

**Affiliations:** 1 Guangxi Engineering Center in Biomedical Material for Tissue and Organ Regeneration, Guangxi Medical University, Nanning, China; 2 The Collaborative Innovation Center of Guangxi Biological Medicine, Guangxi Medical University, Nanning, China; 3 Department of Spine Surgery, The Third Affiliated Hospital of Guangxi Medical University, Nanning, China; 4 Department of Orthopaedics Trauma and Hand Surgery, The First Affiliated Hospital of Guangxi Medical University, Nanning, China; 5 Department of Cell Biology & Genetics, School of Premedical Sciences, Guangxi Medical University, Nanning, China; 6 The Medical and Scientific Research Center, Guangxi Medical University, Nanning, China; 7 Guangxi Key Laboratory of Regenerative Medicine, Guangxi Medical University, Nanning, China

## Abstract

Salidriside (SDS), a phenylpropanoid glycoside derived from *Rhodiola rosea L*, has been shown to be neuroprotective in many studies, which may be promising in nerve recovery. In this study, the neuroprotective effects of SDS on engineered nerve constructed by Schwann cells (SCs) and Poly (lactic-co-glycolic acid) (PLGA) were studied *in vitro*. We further investigated the effect of combinational therapy of SDS and PLGA/SCs based tissue engineering on peripheral nerve regeneration based on the rat model of nerve injury by sciatic transection. The results showed that SDS dramatically enhanced the proliferation and function of SCs. The underlying mechanism may be that SDS affects SCs growth through the modulation of neurotrophic factors (BDNF, GDNF and CNTF). 12 weeks after implantation with a 12 mm gap of sciatic nerve injury, SDS-PLGA/SCs achieved satisfying outcomes of nerve regeneration, as evidenced by morphological and functional improvements upon therapy by SDS, PLGA/SCs or direct suture group assessed by sciatic function index, nerve conduction assay, HE staining and immunohistochemical analysis. Our results demonstrated the significant role of introducing SDS into neural tissue engineering to promote nerve regeneration.

Peripheral nerve injuries caused by accidental trauma, acute compression, iatrogenic injury or hematomas are cosmopolitan health concerns, which may lead to the disruption of myelin sheaths and axons^1^. Cracked nerve larger than 1–2 cm require bridging strategies for repair^2^. In clinical practice, autologous nerve grafting remains the gold standard for the restoration of structural and functional nerve regeneration. Autografts have the advantages of low immunological rejection and morphologically native structure model which extremely promote the regeneration of the cracked nerve. However, autografting has drawbacks such as loss of sensory and motor function at donor site, formation of potential painful neuromas and limitations of the donor length^3^,^4^. Thus, innovative and multi-disciplinary strategies for therapeutic interventions are of pivotal importance to be developed.

One strategy for nerve repair is neural tissue engineering which involves a supportive matrix as nerve guide conduit, the interactive triad of responsive cells and bioactive molecules promoting differentiation to produce a perfectly suited three-dimensional nerve graft for implantation to the lesion site. In this regard, scaffold design is of importance as scaffold creates the environment for controlling cell behavior, cell attachment and proliferation. And it guides the direction of newly sprouted axons and migration of cells towards or outwards the graft^5^. Poly (lactic-co-glycolic acid) (PLGA) is one of the most successfully used biodegradable polymers which hydrolyzes to metabolite monomers, lactic acid and glycolic acid. As one of the few biomaterials approved by the United States Food and Drug Administration (FDA) and European Medicine Agency (EMA) for experimental and clinical application, PLGA has broad utility in drug delivery, artificial catheter and tissue engineering^6^,^7^. Due to the easy controlling of mechanical properties and biodegradation, PLGA is one of the top biodegradable synthetic polymers used for tissue engineering, especially neural tissue engineering^8^. It has been used as carrier for various cells to restore injured nerve^9^,^10^. Among the cells, Schwann cells (SCs) which can stimulate the axonal growth, myelinate regenerating axons and reduce cyst formation and secondary damages to the tissue are promising^11^. The beneficial effects of transplanted Schwann cells have been clearly demonstrated in several animal studies^12^,^13^,^14^. And the role of SCs in the promotion of axon regeneration was confirmed by experiments using the PLGA conduit^15^.

Another important factor in tissue engineering is bioactive molecules which can enhance neurogenesis. However, most bioactive molecules such as growth factors and cytokines are expensive and instable in clinic efficacy, sometimes have side effects^16^. An alternative is the use of plant extracts which always have multi-target and minimum side effects. The key is to find effective plant extracts that are not only harmless, but also effective in axonal regeneration and functional recovery. Rhodioloside (syn. SDS, structure shown in Fig. 1A), a phenylpropanoid glycoside extracted from *Rhodiola rosea L*, a popular plant in traditional medicine in Eastern Europe and Asia, has been reported to have diverse pharmacological properties including antiviral, anticancer, hepatoprotective, antidiabetic and antioxidative effects^17^,^18^,^19^,^20^,^21^. SDS has been shown to be neuroprotective in many studies, which raises the possibility of using SDS as a neuroprotective agent after nerve injuries^22^,^23^,^24^. Sheng QS and *et al*. reported that SDS achieved functionally successful nerve regeneration in the rat as evidenced by walking track analysis, electrophysiological assessment and histological evaluation^22^. Thus, introduction of bioactive chemicals such as SDS in neural tissue engineering may accelerate nerve regeneration.

In the present study, we intended to investigate the neuroprotective effects of SDS on engineered nerve constructed by RSC 96 and PLGA *in vitro* and further the possibility of combining SDS therapy with tissue engineering strategy on peripheral nerve regeneration based on the rat model of nerve injury by sciatic transection.

## Results

### Effects of SDS on RSC 96 *in vitro*

#### Preliminary drug screening and cell cytotoxicity assay

**Figure 1:**
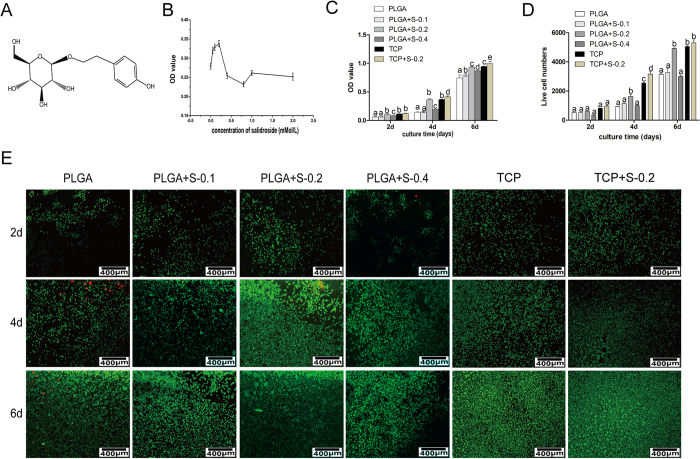
Effects of SDS on RSC 96 via MTT analysis *in vitro*. (**A**) Chemical structure of salidroside; (**B**) Preliminary drug screening analysis of RSC 96 treated on PLGA scaffold with different concentrations of salidroside after 3 days (n = 3, mean ± SEM); (**C**) Proliferative effects of salidroside on RSC96 on PLGA scaffold measured by MTT assay (n = 3, mean ± SEM). Different letters denote significances with P < 0.05 and the same letter shows no significant differences (P ≥ 0.05); (**D**) Quantitative data of the mean number of SCs. Data of each bar are shown as the mean of three independent experiments ± SD. Different letters denote significances with P < 0.05 and the same letter shows no significant differences (P ≥ 0.05); (**E**) Cell viability was measured by FDA/PI staining under microscope. (PLGA means cultured with 0 mM SDS, PLGA+S-0.1 means cultured with 0.1 mM SDS, PLGA+S-0.2 means cultured with 0.2 mM SDS, PLGA+S-0.4 means cultured with 0.4 mM SDS, TCP means cultured on TCP alone, TCP+s-0.2 means cultured with 0.2 mM SDS on TCP).

For preliminary screening, RSC 96 were cultured on the PLGA membrane and treated with SDS in increasing concentrations (0.0125 to 2 Mm) compared to the control group (0 mM). As shown in Fig. 1B, no or very low cytotoxic effect was observed in RSC96 treated with the SDS ranged from 0.0125 to 2 mM. The concentrations of 0.1, 0.2 and 0.4 mM within which a peak value was observed were chosen for further investigation based on the results of preliminary screening.

To determine the cytotoxic effects of SDS on RSC96 growth cultured on PLGA or tissue culture plastic (TCP), 3-(4,5-dimethylthiazol-2-yl)-2,5-diphenyltetrazolium bromide (MTT) was used to analyze cell proliferation of RSC96 in six groups. As shown in Fig. 1C and D, the proliferation of RSC96 is time-dependent in all the groups. Cells treated by SDS of 0.2 increased significantly (P < 0.05) on PLGA compared with other PLGA groups at each time point. Comparatively, TCP can better support the cell proliferation than PLGA, especially in the presence of SDS. Among all the SDS groups, SDS of 0.2 mM increased the cell growth most prominently.

#### Cell viability

Cell viability was determined by FDA and PI, in which viable cells were stained green and dead cells were stained red. As shown in Fig. 1E, more viable cells and less dead cells were presented on PLGA with the addition of SDS of 0.2 mM than other PLGA groups at different culture time, which confirmed the MTT analysis. Cells on TCP treated with SDS proliferated better than on TCP alone, which strongly supports the beneficial effect of salidroside on RSC96 survival.

#### Cell morphology

**Figure 2:**
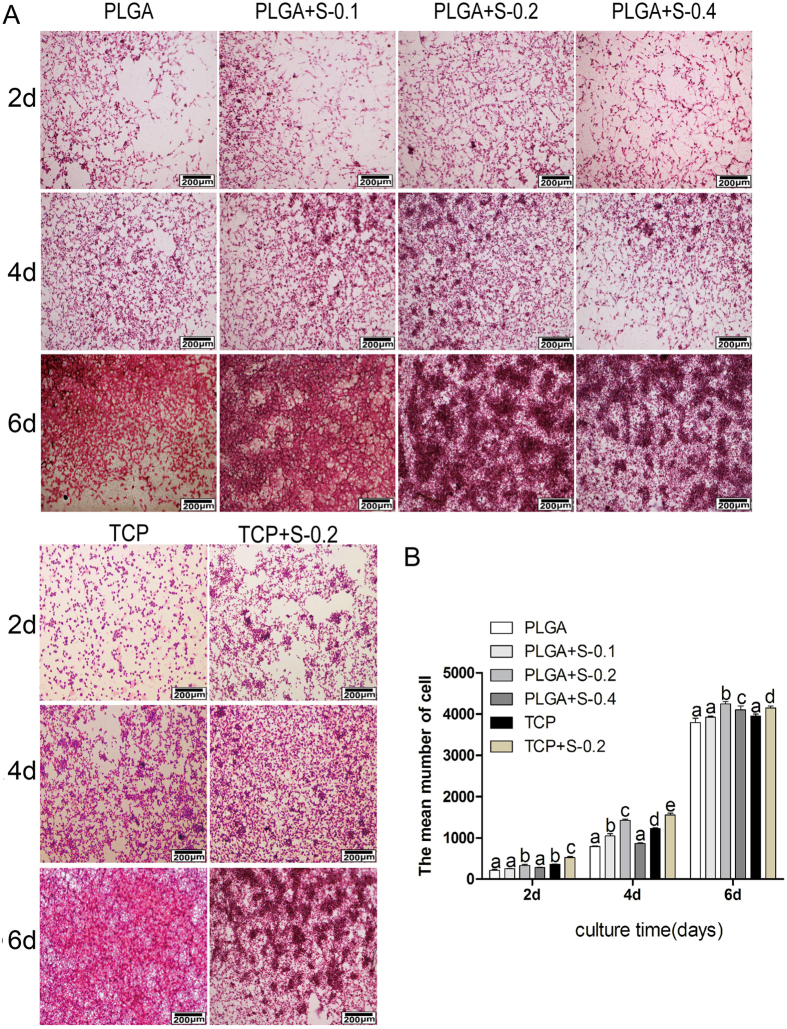
Effects of SDS on RSC 96 via H&E analysis *in vitro*. (**A**) Hematoxylin-eosin staining images showing the morphology of RSC 96 cultured *in vitro* in six groups at 2, 4 and 6 days. (PLGA means cultured with 0 mM SDS, PLGA+S-0.1 means cultured with 0.1 mM SDS, PLGA+S-0.2 means cultured with 0.2 mM SDS, PLGA+S-0.4 means cultured with 0.4 mM SDS, TCP means cultured on TCP alone, TCP+s-0.2 means cultured with 0.2 mM SDS on TCP). (**B**) Quantitative data of the mean number of SCs. Data of each bar are shown as the mean of three independent experiments ± SD. Different letters denote significances with P < 0.05 and the same letter shows no significant differences (P ≥ 0.05).

**Figure 3:**
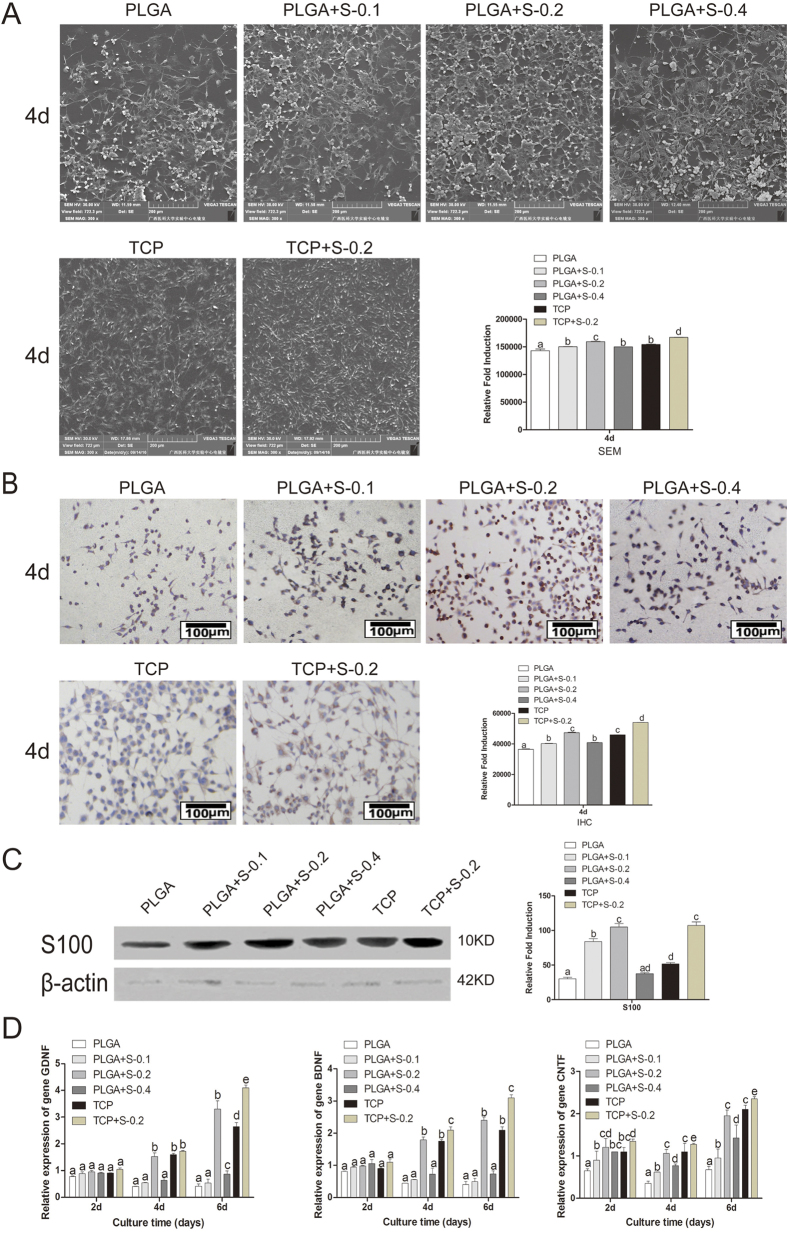
Effects of SDS on RSC 96 via SEM, immunohistochemical analysis, Western Blot assay and gene expression analysis *in vitro.* (**A**) Scanning electron microscopy (SEM) of the cells on scaffolds at 4 days. (PLGA means cultured with 0 mM SDS, PLGA+S-0.1 means cultured with 0.1 mM SDS, PLGA+S-0.2 means cultured with 0.2 mM SDS, PLGA+S-0.4 means cultured with 0.4 mM SDS, TCP means cultured on TCP alone, TCP+s-0.2 means cultured with 0.2 mM SDS on TCP). Statistic analysis of scanning electron microscopy (SEM). Different letters denote significances with P < 0.05 and the same letter shows no significant differences (P ≥ 0.05). (**B**) Immunohistochemical analysis for S-100 protein, RSC 96 showed positive cytoplasmic staining for S-100 at 4 days. (PLGA means cultured with 0 mM SDS, PLGA+S-0.1 means cultured with 0.1 mM SDS, PLGA+S-0.2 means cultured with 0.2 mM SDS, PLGA+S-0.4 means cultured with 0.4 mM SDS, TCP means cultured on TCP alone, TCP+s-0.2 means cultured with 0.2 mM SDS on TCP). Statistic analysis of immunohistochemical analysis. Different letters denote significances with P < 0.05 and the same letter shows no significant differences (P ≥ 0.05). (**C**) Western Blot assay of S-100 protein and quantification of the proteins expression. Full-length blots are presented in supplementary information. Different letters denote significances with P < 0.05 and the same letter shows no significant differences (P ≥ 0.05); (**D**) Gene expression analysis of important neurotrophic factors (GDNF, BDNF and CNTF) by qRT-PCR in six groups at 2, 4 and 6 days. The gene expression levels were analyzed by the 2^−ΔΔCT^ method using GAPDH as the internal control. The data represent the mean of three independent experiments (n = 3, mean ± SEM). Different letters denote significances with P<0.05 and the same letter shows no significant differences (P ≥ 0.05). (PLGA means cultured with 0 mM SDS, PLGA+S-0.1 means cultured with 0.1 mM SDS, PLGA+S-0.2 means cultured with 0.2 mM SDS, PLGA+S-0.4 means cultured with 0.4 mM SDS, TCP means cultured on TCP alone, TCP+s-0.2 means cultured with 0.2 mM SDS on TCP).

Cell morphology and distribution on membrane scaffolds were observed using hematoxylin and eosin kit and scanning electron microscopy (SEM). As showed in Fig. 2A,B and Fig. 3A, the typical component of nerve cells could be clearly observed under the microscope, which indicated that PLGA and SDS of 0.2 mM can better support the cell growth of RSC96 than other PLGA groups. SDS (0.2 mM) alone also promoted cell proliferation, but have no impact on cell morphology.

#### Immunohistochemical analysis

As shown in Fig. 3B, RSC96 showed positive cytoplasmic staining for S-100β which is the marker for RSC96 after cultured for 4 days. The cells cytoplasm on the PLGA membrane were almost stained brown with the round or oval blue nucleus indicating no heteromorphous cells in all groups. More positive staining was shown in RSC96 treated with SDS than untreated counterparts, especially at the concentration of 0.2 mM.

#### Western Blot assay

As shown in Fig. 3C, PLGA and 0.2 mM SDS synergically promoted expression of S100 compared with other PLGA groups. Cells on TCP treated with SDS exhibited higher levels of S100 than TCP group alone.

#### Gene expression

The effect of SDS on RSC96 was further investigated by detecting gene expressions of several important neurotrophic factors, such as glial cell-derived neurotrophic factor (GDNF), brain-derived neurotrophic factor (BDNF), ciliary neurotrophic factor (CNTF). As showed in Fig. 3D, the gene expressions of GDNF, BDNF and CNTF were much higher in the group treated with 0.2 mM SDS compared to other groups in the same substratum, especially at day 4 and day 6 (P < 0.05). The results indicated that SDS of optimal dose stimulated the transcription of GDNF, BDNF and CNTF genes.

### Evaluations for nerve regeneration and recovery of sciatic nerve function

#### Identification of primary SCs for transplantation

**Figure 4:**
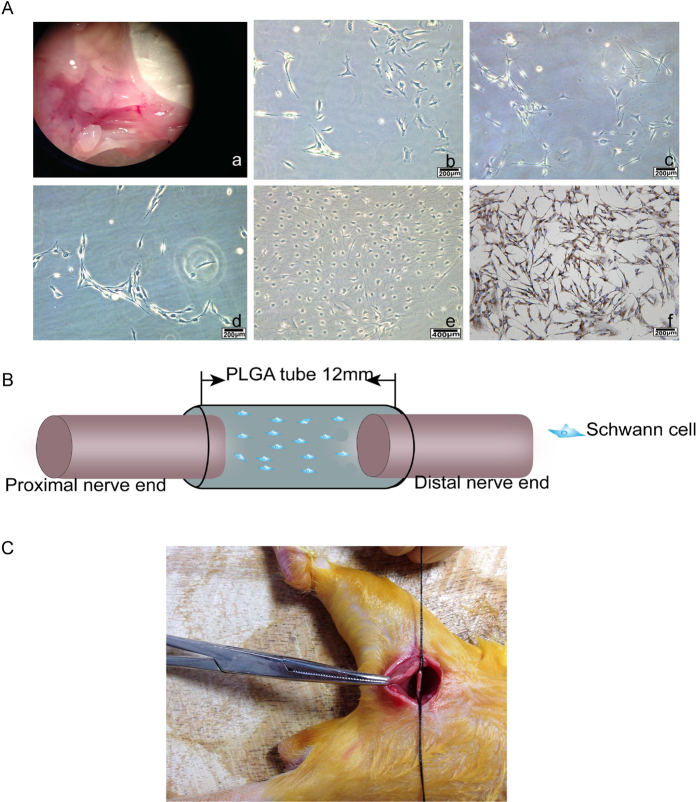
Isolation, purification and identification of primary SCs for transplantation and a rat sciatic nerve model. (**A**) Intraoperative photograph showing the exposure of the sciatic nerve (a) Purification process of SCs at different time points 12 hours (b), 24 hours (c), 72 hours (d), 9days (e) Immunohistochemistry identification of SCs after purification (f). (**B**) Schematic diagram of surgical procedures. (**C**) Photo of the surgical procedure. The sciatic nerve was transected and bridged by the PLGA tube, leaving a 12 mm gap between the proximal and distal nerve stumps.

As showed in Fig. 4A, the purification process of Schwann cells extracted from sciatic nerve (Fig. 4A(a)) were subjected to a series of changes of cell morphology at the time of 12 hours (Fig. 4A(b)), 24 hours (Fig. 4A(c)), 72 hours (Fig. 4A(d)) and for 9 days (Fig. 4A(e)). Cells presented an “end to end, side by side” and other laws of mesh or spiral-shaped arrangement, shaped like a “carpet” (Fig. 4A(f)). Immunohistochemical analysis using antibody of S-100 showed that the purity of the cultured Schwann cells of passage 2 is 98%.

#### Macroscopic findings

**Figure 5:**
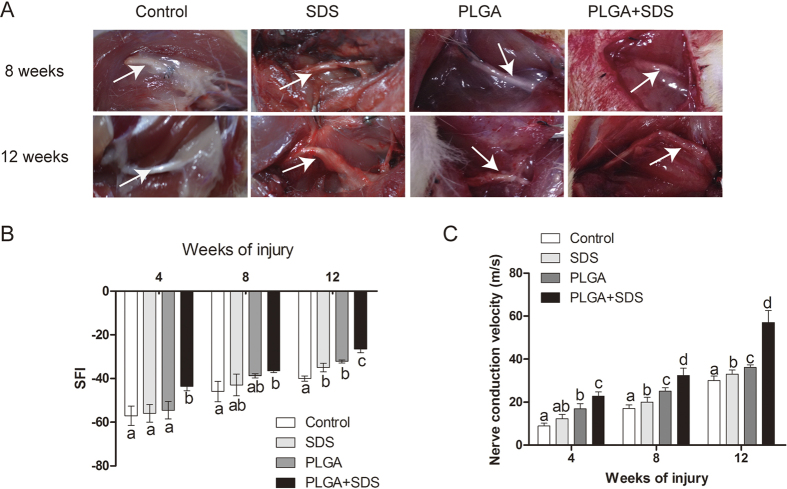
Evaluations for nerve regeneration and recovery of sciatic nerve function. (**A**) Macroscopic findings about PLGA tube in all groups at predetermined time postoperatively (White arrow showing the regenerated nerve); (**B**) The sciatic function index (SFI) values in the four different group measured at the predetermined time postoperatively (n = 3, mean ± SEM). (**C**) Their corresponding nerve conduction velocity (m/s) (n = 3, mean ± SEM), Different letters denote significances with P < 0.05 and the same letter shows no significant differences (P ≥ 0.05).

The model of nerve transected injury was depicted in Fig. 4B and 4C. All animals survived treatment without any serious surgical complications. After the repaired sciatic nerves were harvested, degradation behavior of implants were evaluated by determining the change in macroscopic observations of the harvested samples *in vivo*. As shown in Fig. 5A, degradation of PLGA/SCs and SDS-PLGA/SCs increased gradually from 8 weeks to 12 weeks. No inflammatory reactions could be identified and no neuromas were apparent.

#### Walking track assessment

The parameters used in evaluation of functional restoration in sciatic nerve injuries, assessed by sciatic function index (SFI), are footprint length, toe spread and intermediary toe spread. The index of zero was accepted as normal while −100 indicated complete dysfunction. As the value tends toward zero, a better functional recovery is noted. The SFI values are showed in Fig. 5B. In all the groups, the SFI progressively improved with time but with differing degrees. At 4, 8 weeks post-operation, SDS-PLGA/SCs group showed a significantly better recovery compared to other groups (P < 0.05). There was no significant difference among direct suture group, SDS and PLGA/SCs groups. At 12 weeks post-operation, both SDS and PLGA/SCs group showed significantly better recovery of regenerated nerve compared to control (P < 0.05). In all the groups, SDS-PLGA/SCs showed the best performance, which demonstrated the significant role of introducing SDS into neural tissue engineering in promoting nerve regeneration.

#### Nerve conduction study

Electrophysiological assessment was performed in all groups at 4, 8, 12 weeks. The results of the electrophysiological evaluation are summarized in Fig. 5C. The motor nerve conduction velocity (NCV) in SDS-PLGA/SCs group was significantly faster than the other groups at 4, 8, 12 weeks (P < 0.05), which indicates that combinational therapy of SDS and PLGA based tissue engineering is beneficial for axonal regeneration in rats with transected sciatic nerves.

#### Immunofluorescence staining

**Figure 6:**
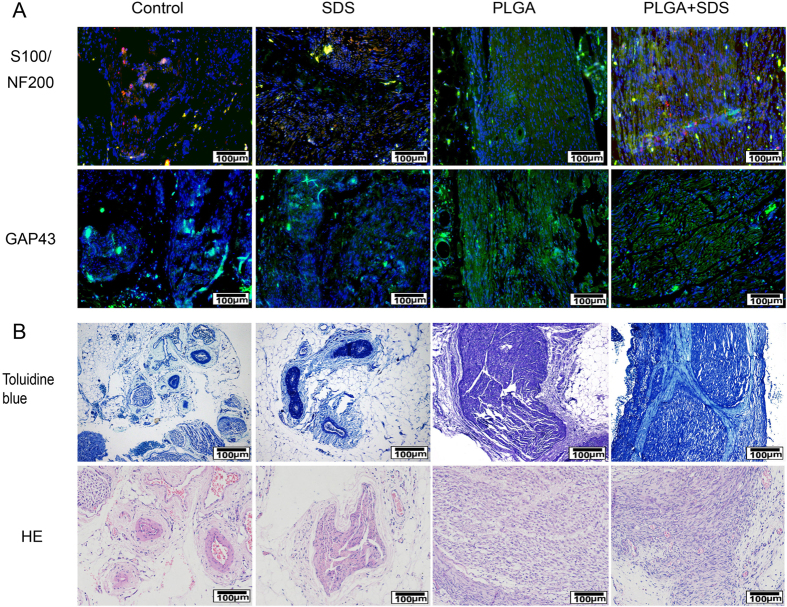
Evaluations for nerve regeneration and recovery of sciatic nerve function. (**A**) Representative longitudinal sections of sciatic nerve at 12 weeks (Immunofluorescence staining of anti-S-100 (red), anti-NF200 (green) and anti-GAP43 (green). The nuclei were stained by DAPI (blue)); (**B**) Representative transverse sections of sciatic nerve at 12 weeks (Hematoxylin and eosin staining and toluidibe blue).

As shown in Fig. 6A, the immunofluorescence findings revealed that more intense positive staining for S-100 (red), NF200 (green) and GAP43 (green) were present in the longitudinal sections of regenerated nerve segments in SDS-PLGA/SCs groups than others at 12 weeks. We also found that two sciatic nerve stumps were reconnected better in SDS-PLGA/SCs than those in other groups. Comparatively, both PLGA and SDS groups showed relatively more positive staining than control.

#### Histological analysis

The promotion effect of SDS and PLGA/SCs constructs on axonal regeneration was further assessed by HE and toluidine blue staining. As shown in Fig. 6B, Massive Schwann cells were observed with ordered arrangement in the SDS-PLGA/SCs group. SDS-PLGA/SCs group achieve better nerve restoration compared to the direct suture group, SDS group and PLGA/SCs group. These observations illustrated that accompanied with nerve regeneration by tissue engineering technique, SDS also accelerate recovery by exerting neuroprotective effects on nerve injury.

### Discuss

*Rhodiola rosea L* has long been used as an adaptogen traditional Chinese medicine. SDS, a phenylpropanoid glycoside extracted from *Rhodiola rosea L*, has been shown to be neuroprotective in many studies^22^,^23^,^24^. The present study was intended to demonstrate the effect of SDS on RSC 96 cultured on PLGA film *in vitro* and further the combinational therapy on nerve regeneration after nerve transection injury in rats.

In the present study, cell proliferation and growth were significantly enhanced in 0.2 mM SDS group compared with untreated counterparts, as evidenced by preliminary drug screening (Fig. 1B), cell cytotoxicity assay (Fig. 1C), cell viability assay (Fig. 1E) and morphological evaluation (Fig. 2). S100, which is Schwann cell marker, was also elevated at protein level (Fig. 3C). In correspondence with the immunohistochemical staining, the upregulated expression of BDNF, GDNF and CNTF (Fig. 3D) indicated the positive effect of SDS on SCs *in vitro*. It has been reported that the expression of several neurotrophic factors secreted by SCs such as BDNF, GDNF and CNTF) is related to the regeneration of axons^25^,^26^,^27^. This may corroborate that SDS exerts effect on SCs and nerve regeneration by regulating the neurotrophic factors. The present study also highlights the possibility of promoting nerve regeneration in cellular nerve grafts through SDS-increased NCV, SFI, neurotrophin secretion. NF200 and GAP43 are regeneration-associated markers important in the growth and functional recovery of damaged sensory and motor axons^28^,^29^, which were also slightly increased by SDS administration.

Over the past century, a series of research has shown the feasibility of natural and synthetic polymers to support neural tissue engineering development and their capacity to conduct the repair of nerve tissue^30^. PLGA is one of the most successfully therapeutic strategies in peripheral nerve repair because of its biocompatibility and biodegradability. In this study, during the process of nerve regeneration, the PLGA tube was stably maintained its tube structure without collapse which is an important factor for axonal growth (Fig. 5A). Strategies of using PLGA carried with SCs were useful for the regeneration of injured nerve, which is superior to that by suture. Combination of PLGA/SCs and SDS is improved over the individual therapy, which altogether contributes to ordered arrangement and functional recovery of nerve. In the combinational therapy, PLGA/SCs may provide the engineered nerve for regeneration of myelinated nerve fibers and SDS may facilitate the therapeutic effect by promoting the expression of neurotrophic factors and regeneration-associated markers. Thus, a synergistic effect was produced by the combination of nerve tissue engineering and drug therapy to accelerate the nerve regeneration.

In conclusion, this study demonstrated that SDS has a positive effect on cell adhesion, proliferation and phenotype maintenance of SCs cultured on PLGA *in vitro*. Combinational therapy by SDS administration and neural tissue engineering using SCs seeded PLGA can greatly enhance the regeneration and functional recovery of myelinated nerve fibers. This study may provide reference for clinical application.

## Materials and Methods

### Preparation of materials

SDS (Chengdu Best Reagent Co.,China) powder was dissolved in 0.2% DMSO and prepared as a stock solution with the final concentration of 2 mM. The stock solution was diluted with culture medium immediately prior to treatment. PLGA (lactic to glycolic acid mol ratio, 75:25; Mw 50,000) scaffold was purchased from Ji nan Daigang Biomaterial Co., Ltd, China.

### Effects of SDS on RSC 96 *in vitro*

#### Cell culture

RSC96 were purchased from China Center for Type Culture Collection (CCTCC) and was cultured in Dulbecco’s Modified Eagle’s medium (DMEM):F12 = 1:1 (Thermo Fisher Beijing, China) supplemented with 10% fetal bovine serum (Hangzhou Sijiqing Biological Engineering Materials Co., Ltd) and 1% of penicillin/streptomycin solution (penicillin 100 U/mL, streptomycin 100 U/mL) in incubator at 37 °C with 5% CO_2._

#### Preliminary drug screening and cell cytotoxicity assay

Preliminary screening and cytotoxicity analysis were assessed using the 3-(4,5-dimethylthiazol-2-yl)-2,5-diphenyltetrazolium bromide (MTT, Sigma-Aldrich) method. For preliminary screening, cells were cultured with various concentrations of SDS (0.0125–0.8 mM) for 3d. Optimal concentrations of SDS were chosen for further study based on the results of preliminary screening. For cell cytotoxicity assay, RSC 96 were seeded on PLGA film at a density of 1 × 10^4^ cells/cm^2^ with the addition of SDS (0, 0.1, 0.2, 0.4 respectively) or seeded on TCP with or without SDS (0.2 Mm) for 2, 4 and 6 days. Briefly, MTT (0.5 mg/ml) was added to each well. After incubation for 4 hours, dimethyl sulfoxide (DMSO, Sigma-Aldrich) was added. The spectrometric absorbance at 570 nm was read using an enzyme-labeled instrument (Thermo Fisher Scientific, UK). Preliminary screening and cytotoxicity assays were performed in triplicate.

#### Cell viability assay

RSC 96 were cultured on PLGA with SDS of 0 mM, 0.1 mM, 0.2 mM and 0.4 mM respectively or cultured on TCP with or without SDS (0.2 Mm) for 2, 4, 6 days. Cell viability was examined by fluorescein diacetate (FDA; Sigma-Aldrich Co., USA) and propidium iodide (PI; Sigma-Aldrich Co., USA). Images were visualized with a laser scanning confocal microscope (Nikon, Japan).

#### Cell morphological analysi

RSC 96 were cultured on PLGA with SDS of 0 mM, 0.1 mM, 0.2 mM and 0.4 mM respectively or cultured on TCP with or without SDS (0.2 Mm) for 2, 4, 6days. Cells were stained using hematoxylin and eosin kit (HE, JianCheng Biotech, China). Images were photographed by an inverted phase contrast microscope (Zeiss Corporation, German).

#### SEM of the cells on scaffolds

RSC 96 cultured on PLGA film or TCP were fixed in 2.5% glutaraldehyde (Merck KGaA, Darmstadt, Germany) for 24 hours. Samples were dehydrated in ascending concentrations of ethanol and then air-dried for 24 hours. They were mounted on stubs, coated with gold-palladium and examined by SEM (Hitachi, Japan).

#### Immunohistochemical analysis

Immunohistochemical staining of S100 was performed following the manufacturer’s instructions. Rabbit anti-rat IgG antibody (1:100) to S100β (S-100; Boster company, Wuhan, China) was added and incubated at room temperature for 2 hours. HRP Polymer-anti-Rabbit IgG was added for 30 min. Diaminobenzadine (DAB) was added to visualize primary antibody staining and counterstained with hematoxylin for 20 seconds. The mounted slides were observed by using a microscope.

#### Western Blot assay

Cell proteins were extracted using RIPA Lysis Buffer (Beyotime, China) and phenylmethanesulfonyl fluoride (PMSF) (Beyotime, China). Briefly, protein were subjected to electrophoresis in the presence of sodium dodecyl sulfate (SDS-PAGE), transferred PVDF membrane (Millipore, USA) and incubated overnight at 4 °C with the following antibodies: anti-S100 and anti-β-actin (Boster company, Wuhan, China). The membranes were incubated with Alexa Fluor 790 dye-conjugated secondary antibodies (Invitrogen, USA) for 1.5 h. Immuno-reactive bands were detected by visualized by an Odyssey Infrared Imaging System (LI-COR) according to the manufacturer’s instructions.

#### Real-time quantitative RT-PCR

The gene expressions of GDNF, BDNF and CNTF were detected by reverse transcription-quantitative polymerase chain reaction (RT-qPCR). Total RNA was extracted from SCs using Trizol reagent (Applied Biosystems, Carlsbad, CA, USA/Ambion, Austin, TX, USA). An equal amount of RNA (300 ng) was used as a template and was reverse-transcribed to complementary DNA (cDNA) using a PrimeScript RT reagent Kit with gDNA Eraser (Takara, Dalian). cDNA was amplified by using an SYBR-Green mix kit (Roche company, Berlin, Germany). The primers were shown in Table 1. For real-time quantitative RT-PCR, the procedures were performed using FastStart Universal SYBR Green Master (Roche, US) on a Master cycle reprealplex 4 system (Eppendorf, Germany). All reactions were run in triplicate for each gene. The relative expression of mRNAs was calculated using the comparative 2^−ΔΔCt^ method and normalized against GAPDH.

### A rat sciatic nerve model *in vivo* study

#### Isolation, purification and expansion of primary SCs for transplantation

Sciatic nerves were aseptically removed from 8 neonatal (1–2d) Sprague-Dawley (SD) rats which were purchased from the Center of Experimental Animals of Guangxi Medical University (Nanning, China). Operations were performed under a dissecting microscope (Olympus, Japan). Schwann cells were isolated from sciatic nerves isolated as described^31^. Briefly, sciatic nerves were desheathed, minced, incubated in DMEM (Thermo Fisher Beijing, China) with 0.03% collagenase type I (Gibco, USA) for 45 min and then incomplete saline solution with 0.25% trypsin (Sigma, USA) for 15 min. After triturated though a fire-polished siliconized pasture pipette, the samples were washed twice with DMEM containing10% fetal bovine serum. After centrifugation, the SCs were re-suspended. Culture medium was changed every 48 h. Cell of passage 2 at 80–90% confluence were used for further study. Cells were divided into two groups: PLGA and PLGA/SDS groups. In PLGA and PLGA/SDS groups, cells were seeded on PLGA film for 6 days. For PLGA/SDS group, cells were cultured with the addition of 0.2 mM SDS which was chosen from *vitro* study.

#### Animals and surgery

Adult male SD rats (weighing 250–300 g) were used in our study provided by the center of experimental animals of Guangxi Medical University and approved by the Ethics Committee of Guangxi Medical University (Nanning, China; number 20140307A; 20140307B). All methods were performed in accordance with the relevant guidelines and regulations. The rats were randomly divided into four groups including direct suture group (Control), SDS group, PLGA/SCs group and SDS-PLGA/SCs group with 20 rats per group. In both PLGA/SCs and SDS-PLGA/SCs groups, PLGA films seeded with primary SCs were prepared as hollow tubes with the diameter of 2 mm and length of 12 mm before implantation. The animals were weighed and anesthetized with an intraperitoneal injection of pentobarbital (30 mg/kg). The left sciatic nerves were freed from the adjacent muscles by an incision extending from the greater trochanter to the midcalf distally. The sciatic nerve was cut off from its proximal to a distal segment. In direct suture group the nerve were sutured end to end. In both the PLGA/SCs and SDS-PLGA/SCs groups, the 12-mm conduits were placed into this nerve gap using 10–0 nylon sutures by microsurgical technique with each nerve end sutured into the conduit about 1 mm, leaving a nerve gap of approximately 10 mm. Finally, the skin wound was closed with 3–0 silk sutures. For SDS group and SDS-PLGA/SCs group, SDS solution (dose of 60 mg/kg)^32^ was injected intramuscularly in SD rats from day 3 after surgical operation and continuously injected every day for 21 days.

#### Sciatic function index (SFI)

Walking track analysis, an indirect method to measure function of muscle after reinnervation, was performed monthly for 3 months. The SFI was calculated as previously described according to the following formula^33^:


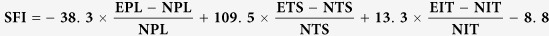


Where EPL is the experimental leg paw length, NPL is the normal leg paw length, ETS is the experimental leg toe spread between the first and fifth toes, NTS is the normal leg toe spread, EIT is the experimental leg intermediate toe spread between the second and the fourth toes and NIT is the normal leg intermediate toe spread. The SFI value varies from 0 to −100, with 0 corresponding to normal function and −100 to total impairment of the sciatic nerve function.

#### Sciatic electrophysiological assessment

The electrophysiological assessment was performed at 4, 8 and 12 weeks after surgery. A bipolar stimulating electrode was contacted with the nerve proximal and the distal to the suture sites respectively. A recording electrode was placed in the gastrocnemius muscle to record electrical activity. The NCV was calculated from the distance and the latency between the distal and proximal stimulated sites.

#### Immunofluorescence staining

The animals were sacrificed and 2 cm of the sciatic nerves rapidly dissected at 12 week. In this study, antibodies to S-100, NF200 and GAP43 (Boster company, Wuhan, China) were used as markers for SCs and new regenerating fiber. Secondary antibodies used were FITC-conjugated goat anti-mouse (1:32, Boster company, Wuhan, China) and Cy3-conjugated goat anti-rabbit antibodies (1:50, Boster company, Wuhan, China). A mounting medium with DAPI (Boster company, Wuhan, China) was used to counterstain the cell nuclei. The slides were examined under a fluorescence microscope.

#### Histological analysis

Twelve weeks post-transplantation. specimens were dehydrated and embedded in paraffin according to standard protocols. Semi-thin sections were stained with HE staining kit (JianCheng Biotech, China) or toluidine blue (JianCheng Biotech, China). Images were photographed by an inverted phase contrast microscope.

#### Statistical analysis

The data were presented as the means ± SD. One-way analysis of variance (ANOVA) was used to compare the means among different groups and Tukey test was used in the post hoc multiple comparisons. The differences were considered statistically significant at p < 0.05.

## Additional Information

**How to cite this article**: Liu, H. *et al*. Salidroside promotes peripheral nerve regeneration based on tissue engineering strategy using Schwann cells and PLGA: *in vitro* and *in vivo. Sci. Rep.* **7**, 39869; doi: 10.1038/srep39869 (2017).

**Publisher's note:** Springer Nature remains neutral with regard to jurisdictional claims in published maps and institutional affiliations.

## Supplementary Material

Supplementary Information

## Figures and Tables

**Table 1:** Genes and oligonucleotide primers used in PCR analysis.

Gene	Primer sequence (5′ to 3′)	Length (bp)	Amplicon size (bp)
GDNF	F: AGACCGGATCCGAGGTGC	18	129
	R: TCGAGAAGCCTCTTACCGGC	20	
BDNF	F: TACCTGGATGCCGCAAACAT	20	182
	R: TGGCCTTTTGATACCGGGAC	20	
CNTF	F: ATGGCTTTCGCAGAGCAAAC	20	191
	R: CAACGATCAGTGCTTGCCAC	20	
GAPDH	F: GTCATCATCTCAGCCCCCTC	20	99
	R:GGATGCGTTGCTGACAATCT	20	

PCR: polymerase chain reaction; GDNF: glial cell-derived neurotrophic factor; BDNF: brain-derived neurotrophic factor; CNTF: ciliary neurotrophic factor; F: forward primer; R: reverse primer.
