# Author Correction: Salidroside promotes peripheral nerve regeneration based on tissue engineering strategy using Schwann cells and PLGA: *in vitro* and *in vivo*

**DOI:** 10.1038/s41598-020-71638-y

**Published:** 2020-09-09

**Authors:** Hui Liu, Peizhen Lv, Yongjia Zhu, Huayu Wu, Kun Zhang, Fuben Xu, Li Zheng, Jinmin Zhao

**Affiliations:** 1 Guangxi Engineering Center in Biomedical Material for Tissue and Organ Regeneration, Guangxi Medical University, Nanning, China; 2 The Collaborative Innovation Center of Guangxi Biological Medicine, Guangxi Medical University, Nanning, China; 3 Department of Spine Surgery, The Third Affiliated Hospital of Guangxi Medical University, Nanning, China; 4 Department of Orthopaedics Trauma and Hand Surgery, The First Affiliated Hospital of Guangxi Medical University, Nanning, China; 5 Department of Cell Biology & Genetics, School of Premedical Sciences, Guangxi Medical University, Nanning, China; 6 The Medical and Scientific Research Center, Guangxi Medical University, Nanning, China; 7 Guangxi Key Laboratory of Regenerative Medicine, Guangxi Medical University, Nanning, China

Correction to: *Scientific Reports*, 10.1038/srep39869, published online 05 January 2017

This Article contains errors.

In Figure 1E the image for PLGA+S-0.2 at 4d is incorrect and is duplicating an image for PLGA+S-0.1 at 6d, which was already correct at the time of publication. The correct Figure 1 appears below.

**Figure 1:**
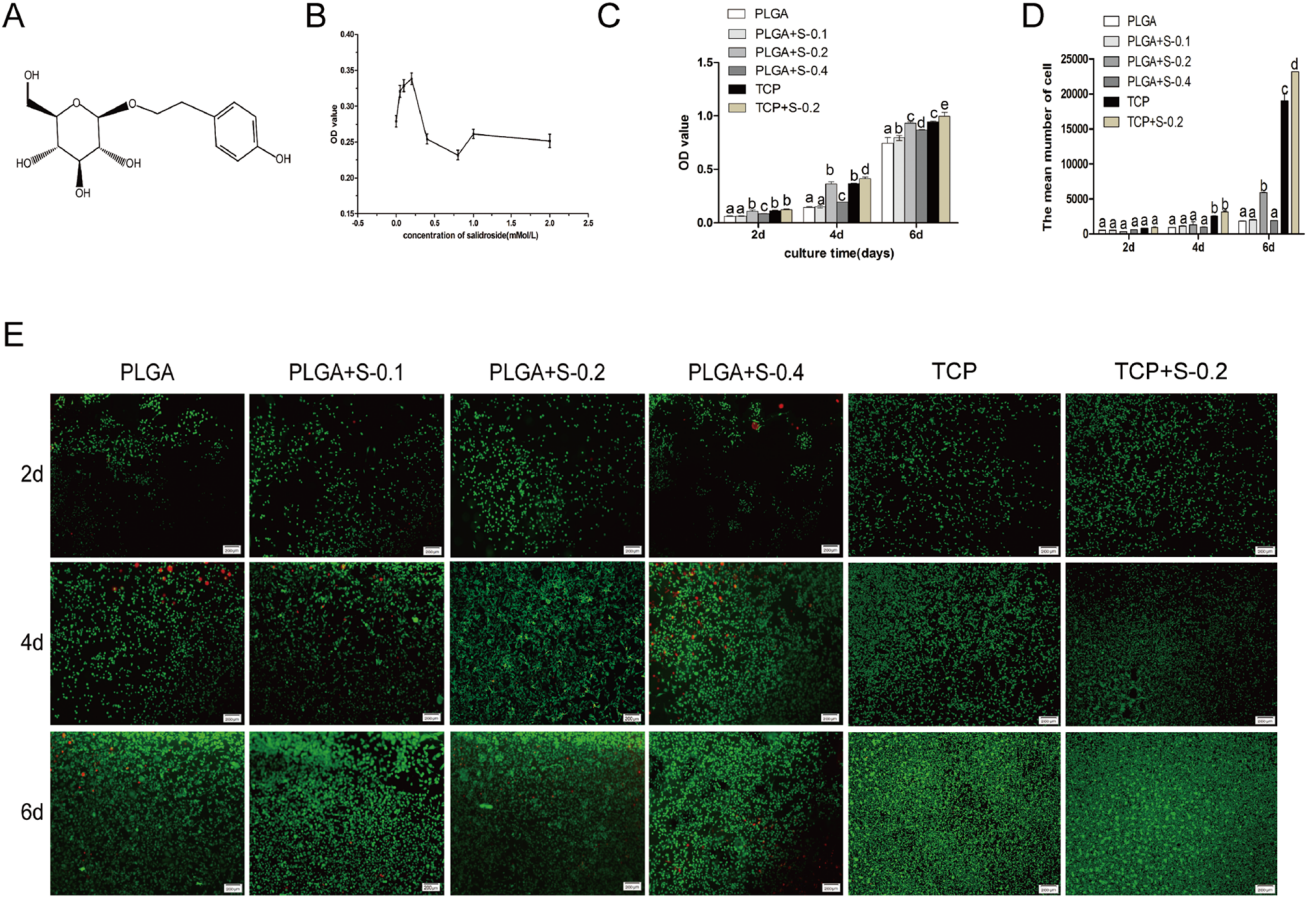
(**A**) Chemical structure of salidroside; (**B**) Preliminary drug screening analysis of RSC 96 treated on PLGA scaffold with different concentrations of salidroside after 3 days (n = 3, mean ± SEM); (**C**) Proliferative effects of salidroside on RSC96 on PLGA scaffold measured by MTT assay (n = 3, mean ± SEM). Different letters denote significances with P < 0.05 and the same letter shows no significant differences (P ≥ 0.05); (**D**) Quantitative data of the mean number of SCs. Data of each bar are shown as the mean of three independent experiments ± SD. Different letters denote significances with P < 0.05 and the same letter shows no significant differences (P ≥ 0.05); (**E**) Cell viability was measured by FDA/PI staining under microscope. (PLGA means cultured with 0 mM SDS, PLGA+S-0.1 means cultured with 0.1 mM SDS, PLGA+S-0.2 means cultured with 0.2 mM SDS, PLGA+S-0.4 means cultured with 0.4 mM SDS, TCP means cultured on TCP alone, TCP+s-0.2 means cultured with 0.2 mM SDS on TCP).

In Figure 2A the images for PLGA at 2d and TCP at 4d are incorrect. The correct Figure 2 appears below.

**Figure 2:**
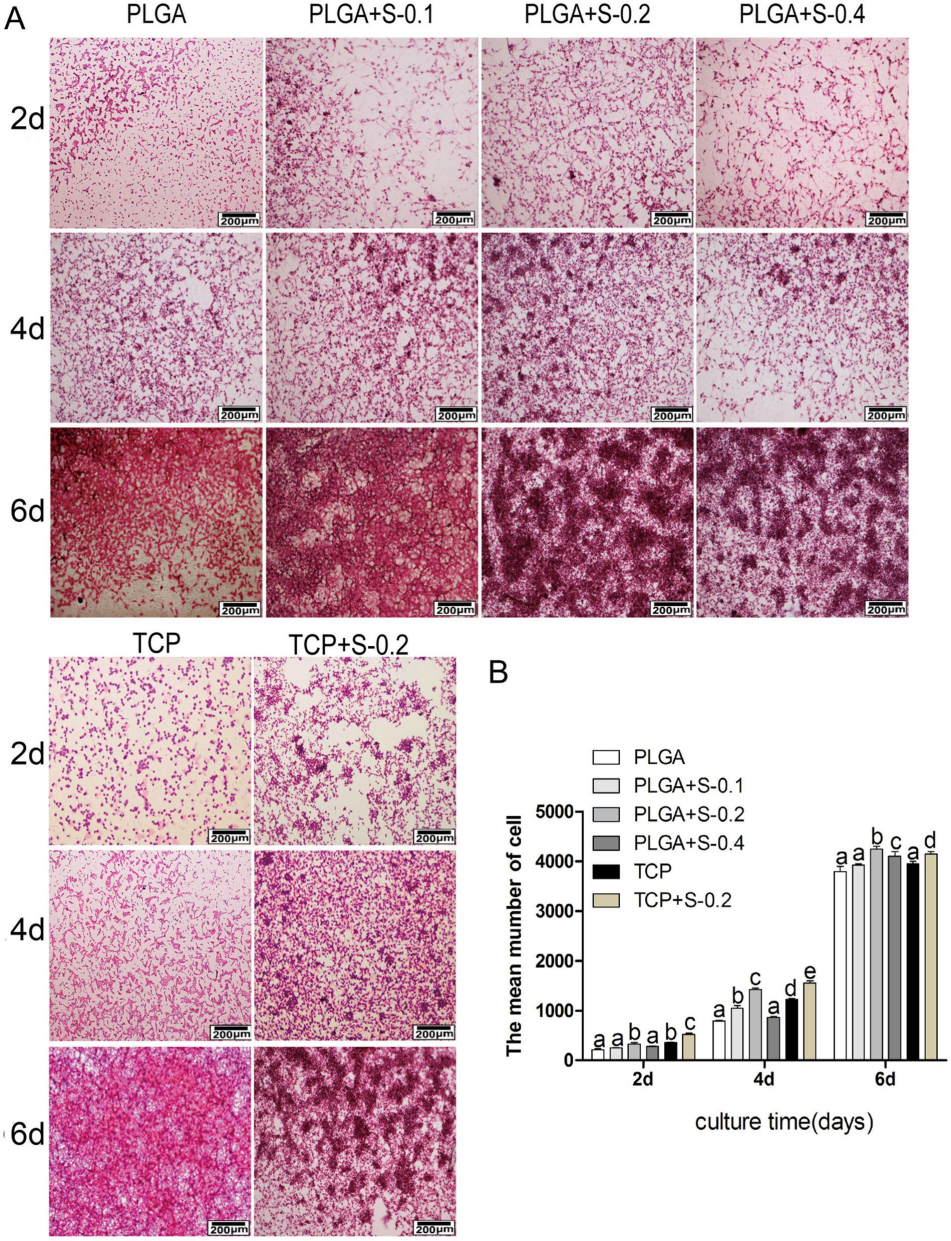
(**A**) Hematoxylin-eosin staining images showing the morphology of RSC 96 cultured in vitro in six groups at 2, 4 and 6 days. (PLGA means cultured with 0 mM SDS, PLGA+S-0.1 means cultured with 0.1 mM SDS, PLGA+S-0.2 means cultured with 0.2 mM SDS, PLGA+S-0.4 means cultured with 0.4 mM SDS, TCP means cultured on TCP alone, TCP+s-0.2 means cultured with 0.2 mM SDS on TCP). (**B**) Quantitative data of the mean number of SCs. Data of each bar are shown as the mean of three independent experiments ± SD. Different letters denote significances with P < 0.05 and the same letter shows no significant differences (P ≥ 0.05).

