# Author Correction: Salidroside promotes peripheral nerve regeneration based on tissue engineering strategy using Schwann cells and PLGA: *in vitro* and *in vivo*

**DOI:** 10.1038/s41598-022-14289-5

**Published:** 2022-06-13

**Authors:** Hui Liu, Peizhen Lv, Yongjia Zhu, Huayu Wu, Kun Zhang, Fuben Xu, Li Zheng, Jinmin Zhao

**Affiliations:** 1 Guangxi Engineering Center in Biomedical Material for Tissue and Organ Regeneration, Guangxi Medical University, Nanning, China; 2 The Collaborative Innovation Center of Guangxi Biological Medicine, Guangxi Medical University, Nanning, China; 3 Department of Spine Surgery, The Third Affiliated Hospital of Guangxi Medical University, Nanning, China; 4 Department of Orthopaedics Trauma and Hand Surgery, The First Affiliated Hospital of Guangxi Medical University, Nanning, China; 5 Department of Cell Biology & Genetics, School of Premedical Sciences, Guangxi Medical University, Nanning, China; 6 The Medical and Scientific Research Center, Guangxi Medical University, Nanning, China; 7 Guangxi Key Laboratory of Regenerative Medicine, Guangxi Medical University, Nanning, China

Correction to: *Scientific Reports* 10.1038/srep39869, published online 05 January 2017

This Article contains errors, that were not addressed in the previous correction^1^.

In Figure 1E, the images for TCP at 2d, PLGA+S-0.1 at 4d, and TCP at 4d are incorrect. A corrected version of Figure 1 and its accompanying legend appear below.

**Figure 1:**
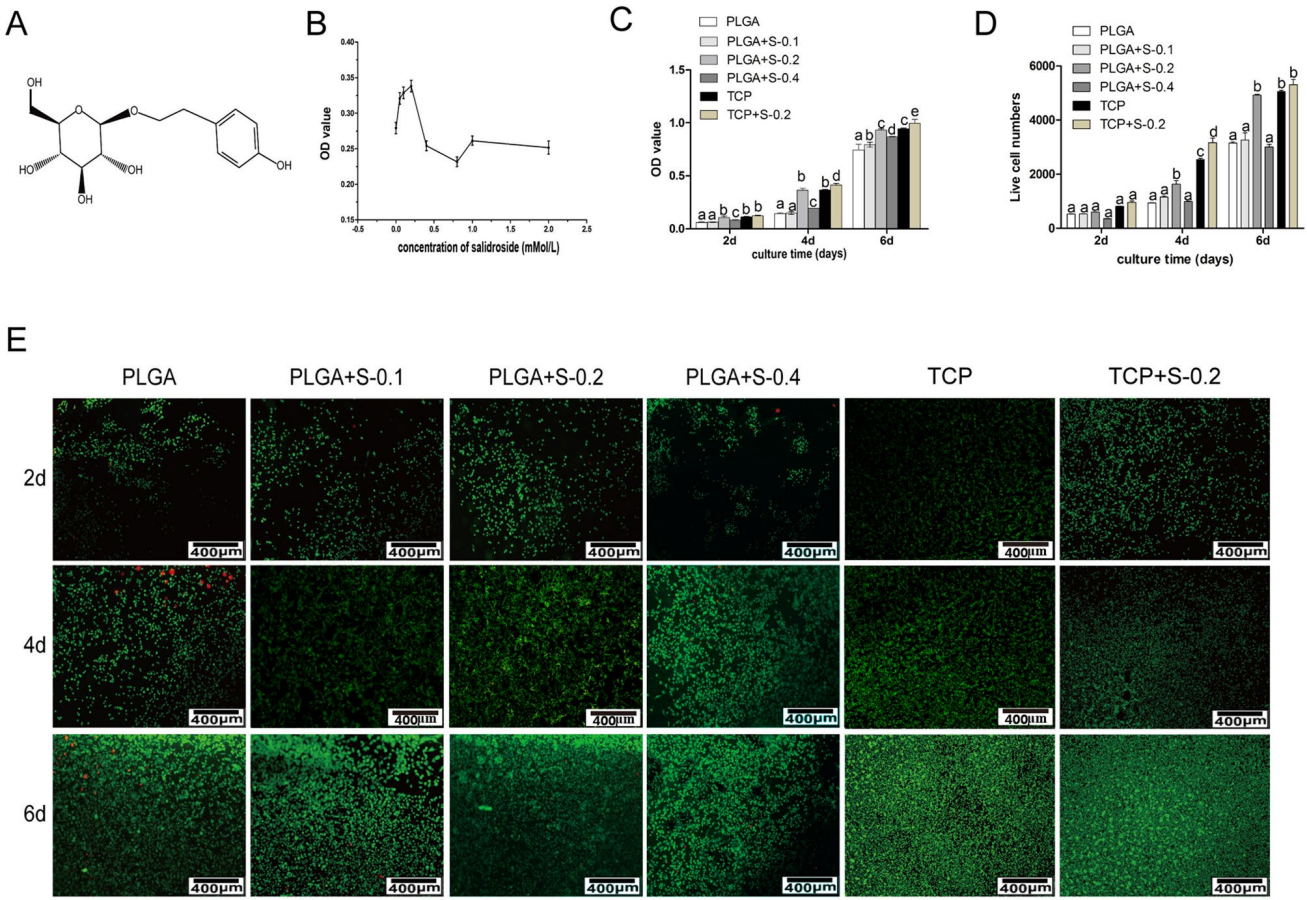
Effects of SDS on RSC 96 via MTT analysis *in vitro*. (**A**) Chemical structure of salidroside; (**B**) Preliminary drug screening analysis of RSC 96 treated on PLGA scaffold with different concentrations of salidroside after 3 days (n = 3, mean ± SEM); (**C**) Proliferative effects of salidroside on RSC96 on PLGA scaffold measured by MTT assay (n = 3, mean ± SEM). Different letters denote significances with P < 0.05 and the same letter shows no significant differences (P ≥ 0.05); (**D**) Quantitative data of the mean number of SCs. Data of each bar are shown as the mean of three independent experiments ± SD. Different letters denote significances with P < 0.05 and the same letter shows no significant differences (P ≥ 0.05); (**E**) Cell viability was measured by FDA/PI staining under microscope. (PLGA means cultured with 0 mM SDS, PLGA+S-0.1 means cultured with 0.1 mM SDS, PLGA+S-0.2 means cultured with 0.2 mM SDS, PLGA+S-0.4 means cultured with 0.4 mM SDS, TCP means cultured on TCP alone, TCP + s-0.2 means cultured with 0.2 mM SDS on TCP).

In Figure 3, the images for PLGA+S-0.1 at 4d and PLGA+S.02 at 4d in Figure 3A and TCP at 4d in Figure 3B are incorrect. A corrected version of Figure 3 and its accompanying legend appear below.

**Figure 3:**
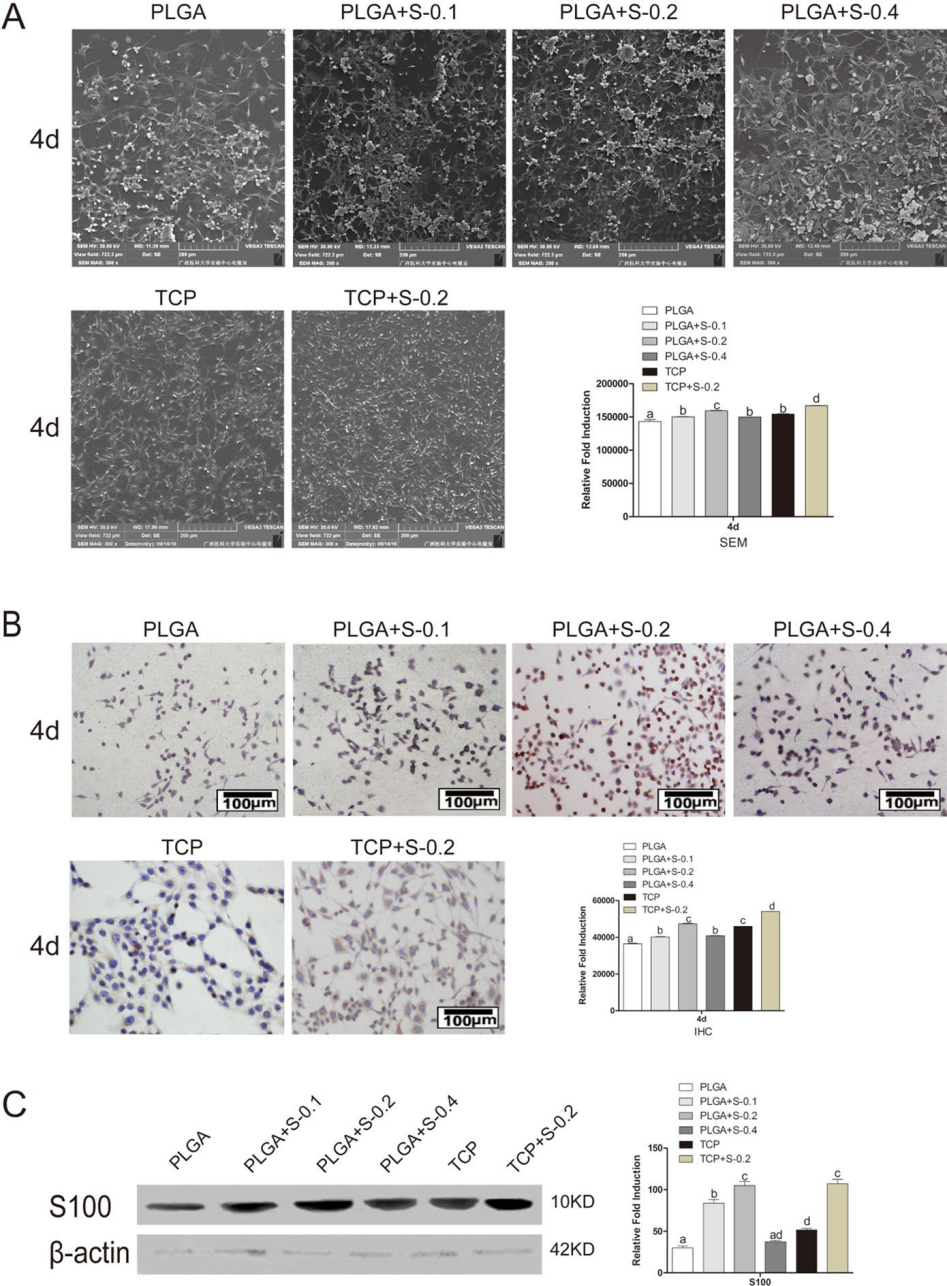

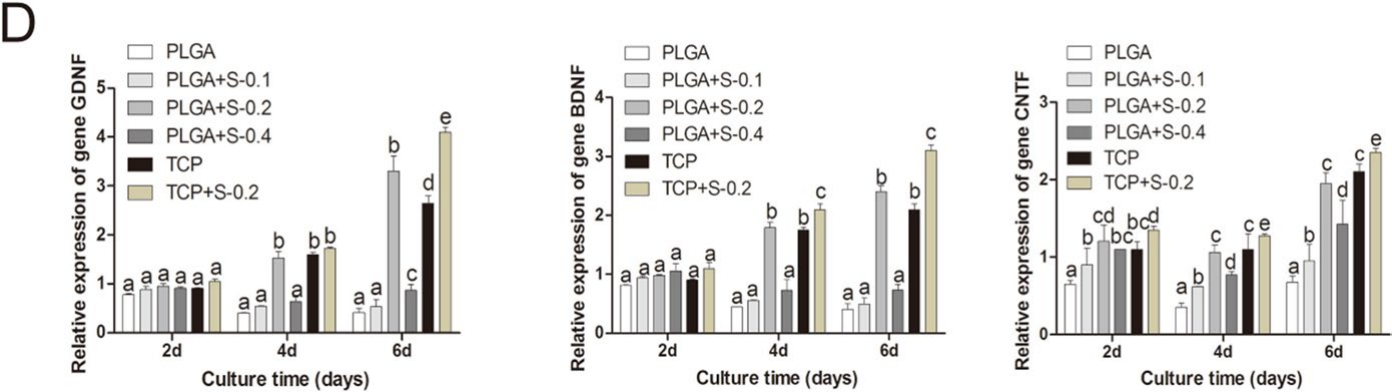
Effects of SDS on RSC 96 via SEM, immunohistochemical analysis, Western Blot assay and gene expression analysis *in vitro.* (**A**) Scanning electron microscopy (SEM) of the cells on scaffolds at 4 days. (PLGA means cultured with 0 mM SDS, PLGA+S-0.1 means cultured with 0.1 mM SDS, PLGA+S-0.2 means cultured with 0.2 mM SDS, PLGA+S-0.4 means cultured with 0.4 mM SDS, TCP means cultured on TCP alone, TCP + s-0.2 means cultured with 0.2 mM SDS on TCP). Statistic analysis of scanning electron microscopy (SEM). Different letters denote significances with P < 0.05 and the same letter shows no significant differences (P ≥ 0.05). (**B**) Immunohistochemical analysis for S-100 protein, RSC 96 showed positive cytoplasmic staining for S-100 at 4 days. (PLGA means cultured with 0 mM SDS, PLGA+S-0.1 means cultured with 0.1 mM SDS, PLGA+S-0.2 means cultured with 0.2 mM SDS, PLGA+S-0.4 means cultured with 0.4 mM SDS, TCP means cultured on TCP alone, TCP + s-0.2 means cultured with 0.2 mM SDS on TCP). Statistic analysis of immunohistochemical analysis. Different letters denote significances with P < 0.05 and the same letter shows no significant differences (P ≥ 0.05). (**C**) Western Blot assay of S-100 protein and quantification of the proteins expression. Full-length blots are presented in supplementary information. Different letters denote significances with P < 0.05 and the same letter shows no significant differences (P ≥ 0.05); (**D**) Gene expression analysis of important neurotrophic factors (GDNF, BDNF and CNTF) by qRT-PCR in six groups at 2, 4 and 6 days. The gene expression levels were analyzed by the 2^−ΔΔCT^ method using GAPDH as the internal control. The data represent the mean of three independent experiments (n = 3, mean ± SEM). Different letters denote significances with P < 0.05 and the same letter shows no significant differences (P ≥ 0.05). (PLGA means cultured with 0 mM SDS, PLGA+S-0.1 means cultured with 0.1 mM SDS, PLGA+S-0.2 means cultured with 0.2 mM SDS, PLGA+S-0.4 means cultured with 0.4 mM SDS, TCP means cultured on TCP alone, TCP + s-0.2 means cultured with 0.2 mM SDS on TCP).
