# Supplementary material for: Salidroside promotes peripheral nerve regeneration based on tissue engineering strategy using Schwann cells and PLGA: in vitro and in vivo
Source: Sci Rep. 2017 Jan 5;7:39869. doi: 10.1038/srep39869 (PMC5213129; doi:10.1038/srep39869)
Supplement: Supplementary Information [file srep39869-s1.pdf]

Salidroside promotes peripheral nerve regeneration based on tissue engineering strategy using Schwann cells and PLGA: *in vitro* and *in vivo*

Hui Liu[1,2+], Peizhen Lv[1, 3+], Yongjia Zhu[4+], Huayu Wu[5], Kun Zhang[1,4], Fuben Xu[1, 6] Li Zheng[1, 2, 6*], Jinmin Zhao[1, 2, 4, 7*]

[1]Guangxi Engineering Center in Biomedical Material for Tissue and Organ Regeneration, Guangxi Medical University, Nanning, China

[2] The Collaborative Innovation Center of Guangxi Biological Medicine, Guangxi Medical University, Nanning, China

[3]Department of Spine Surgery, The Third Affiliated Hospital of Guangxi Medical University, Nanning, China.

[4] Department of Orthopaedics Trauma and Hand Surgery, The First Affiliated Hospital of Guangxi Medical University, Nanning, China.

[5] Department of Cell Biology & Genetics, School of Premedical Sciences, Guangxi Medical University, Nanning, China

[6] The Medical and Scientific Research Center, Guangxi Medical University, Nanning, China

[7] Guangxi Key Laboratory of Regenerative Medicine, Guangxi Medical University, Nanning, China

Hui Liu, Peizhen Lv and Yongjia Zhu contributed equally to this work.

* Co-Corresponding authors.

Li Zheng: Tel: +86-07715358132. Fax: +86-07715350975. Email address: zhengli224@163.com

Jinmin Zhao: Tel: +86-07715350189. Fax: +86-07715350189. Email address: zhaojinmin@126.com

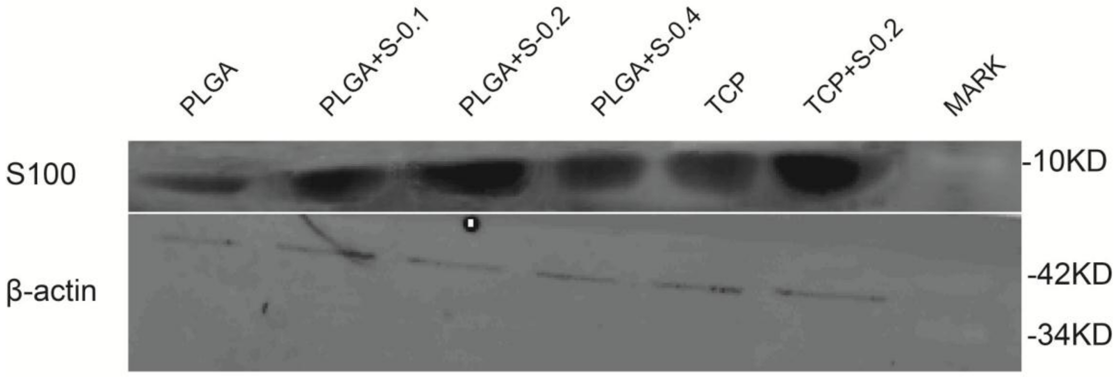

Legend: Full-length blots of S-100 protein in Figure 3C. (PLGA means cultured with 0 mM SDS, PLGA+S-0.1 means cultured with 0.1 mM SDS, PLGA+S-0.2 means cultured with 0.2mM SDS, PLGA+S-0.4 means cultured with 0.4mM SDS, TCP means cultured on TCP alone, TCP+s-0.2 means cultured with 0.2mM SDS on TCP).
